# Role of Cox-2 in Vascular Inflammation: An Experimental Model of Metabolic Syndrome

**DOI:** 10.1155/2013/513251

**Published:** 2013-02-14

**Authors:** Nicolás F. Renna, Emiliano R. Diez, Carina Lembo, Roberto M. Miatello

**Affiliations:** ^1^Department of Pathology, School of Medicine, National University of Cuyo, Avenida Libertador No. 80, Centro Universitario, CP 5500 Mendoza, Argentina; ^2^Institute of Experimental Medicine and Biology of Cuyo (IMBECU)-CONICET, Mendoza, Argentina; ^3^Department of Morphophysiology, School of Medicine, National University of Cuyo, Avenida Libertador No. 80, Centro Universitario, CP 5500 Mendoza, Argentina

## Abstract

The objective of this work was to demonstrate the role of COX-2 enzyme at the vascular in experimental model of metabolic syndrome. SHR male WKY rats were employed; they were distributed in 8 groups (*n* = 8 each): control (W); W + L: WKY rats receiving 20 mg/kg of lumiracoxib by intraesophageal administration; SHR; SHR + L: SHR + 20 mg/kg of lumiracoxib by intraesophageal administration; Fructose-Fed Rats (FFR): WKY rats receiving 10% (w/v) fructose solution in drinking water during all 12 weeks; FFR + L: FFR + 20 mg/kg of lumiracoxib by intraesophageal administration; Fructose-Fed Hypertensive Rats (FFHR): SHR receiving 10% (w/v) fructose solution in drinking water during all 12 weeks; and FFHR + L: FFHR + 20 mg/kg of lumiracoxib by intraesophageal administration. Metabolic variables, blood pressure, morphometric variables, and oxidative stress variables were evaluated; also MMP-2 and MMP-9 (collagenases), VCAM-1, and NF-**κ**B by Westernblot or IFI were evaluated. FFHR presented all variables of metabolic syndrome; there was also an increase in oxidative stress variables; vascular remodeling and left ventricular hypertrophy were evidenced along with a significant increase in the expression of the mentioned proinflammatory molecules and increased activity and expression of collagenase. Lumiracoxib was able to reverse vascular remodeling changes and inflammation, demonstrating the involvement of COX-2 in the pathophysiology of vascular remodeling in this experimental model.

## 1. Introduction

The traditional view of atherosclerosis as a lipid storage disease falls apart against the large and growing evidence that inflammation is at the center of all stages of the disease, from the initial injury until the final stage of thrombotic complications that compromise blood flow. Advances in the understanding of vascular inflammation have resulted in a radical change in the way vascular diseases are approached. With increased awareness of the active role of the vessel and its complex interactions with cytokines and immune cells, this concept unites disorders previously thought be different. Understanding atherosclerosis as a vascular inflammation disease is the basis of a new approach for risk stratification and treatment [1].

Matrix metalloproteinases (MMPs) play an important role in maintaining homeostasis of extracellular structures. MMPs are induced by cytokines and by cell-cell and cell-matrix interactions. Examples of the increased presence of MMPs in clinical pathology are the SCA, specifically in the vulnerable region of the plaque [2]. Exposure to oxidized low density lipoproteins (ox-LDL) or TNF-*α* induces the expression of MT3-MMP, an MMP expressed in the atherosclerotic plaque of macrophages [3]. 

C-reactive protein (CRP) presents a rapid and dramatic response to an inflammatory stimulus. Ultrasensitive C-reactive protein (hsCRP) has a very important role in the detection of vascular inflammation and cardiovascular risk prediction. There is evidence that CRP is involved in atherosclerosis, especially at its beginning. Proinflammatory cytokines production in monocytes and macrophages is stimulated by PCR [4]. CAMs expression is mediated by CRP, allowing the increase of leukocyte adhesion and migration [5–7].

Spontaneously hypertensive rats (SHR) provide a model of genetic hypertension that allows studying essential hypertension. By administrating carbohydrate diets to rats it is possible to induce insulin resistance, hyperinsulinemia, dyslipidemia, and hypertension. Fructose-Fed-Rats (FFR) provides a useful experimental model for the study of the interaction factors shaping metabolic syndrome. This combined model (FFHR) is representative of hypertensive individuals who eat a modern Western diet rich in refined sugars. Some authors postulate this dual model as the most appropriate for extrapolating results to human models [8, 9].

This experimental model has proved in previous works its utility for the study of the interaction factors shaping insulin resistance syndrome [10], including endothelial dysfunction, the decrease, at the cardiovascular level, of the activity of the endothelial isoform of nitric oxide synthase (eNOS), and the increase in the proliferation of vascular smooth muscle cells [11], and has also provided evidence involving RAS in its pathophysiology [12].

The objective of this work was to demonstrate that vascular inflammation and oxidative stress are involved in the pathophysiologic mechanisms of structural and functional vascular changes (remodeling) associated to the experimental model of metabolic syndrome through administration of lumiracoxib (L) as COX-2 specific anti-inflammatory. 

## 2. Methods

### 2.1. Animals and Experimental Design

All procedures were performed according to institutional guidelines for animal experimentation; protocol was submitted and approved by the Institutional Committee for Laboratory Animal Use and Care (CICUAL) of the School of Medicine, UNCuyo. Thirty-day-old male Wistar Kyoto (WKY) rats and SHR were fed a standard commercial chow diet ad libitum and housed in a room under conditions of controlled temperature (20°C) and humidity, with a 12-hour light/dark cycle during a 12-week experimental period. Lumiracoxib (L) was administrated to respective groups during the last six weeks. Study groups were divided as follows.

(i) Control (W): WKY receiving food and drinking water (DW) ad libitum;
(ii) SHR: receiving food and DW ad libitum;
(iii) Fructose-Fed Rats (FFR): WKY receiving 10% (w/v) fructose (Parafarm, Buenos Aires, Argentina) solution in DW during all 12 weeks;
(iv) Fructose-Fed Hypertensive Rats (FFHR): SHR receiving 10% (w/v) fructose solution in DW during all 12 weeks;
(v) FFR + L:FFR receiving 20 mg/kg L by intraesophageal administration;
(vi) FFHR + L:FFHR receiving 20 mg/kg L by intraesophageal administration.

At the end of the experimental period, rats were anesthetized with sodium pentobarbital (50 mg/Kg ip), blood samples were taken, and arteries and organs were aseptically excised for measurements. 

### 2.2. Systolic Blood Pressure Measurement

Systolic blood pressure (SBP) was monitored indirectly in conscious pre-warmed slightly restrained rats by the tail-cuff method and recorded on a Grass Model 7 polygraph (Grass Instruments Co., Quincy, MA, USA). The rats were trained in the apparatus several times before measurement. 

### 2.3. Biochemical Determinations

#### 2.3.1. HOMA Index and Intraperitoneal Glucose Tolerance Test

Fasting plasma insulin was assayed by ACS:180SE automated chemiluminescence system (Bayer, Germany). Plasma glucose levels were assayed using a commercial colorimetric method (Wiener Lab., Argentina). Homeostasis model assessment (HOMA) was used as an index to measure the degree of insulin resistance; it was calculated using the following formula: (insulin (*μ*U/mL) × glucose (mmol/L)/22.5) [13].

Three days before the end of the experimental period, a glucose tolerance test (GTT) was performed. Rats fasted overnight were slightly anesthetized with pentobarbital, and glucose was administered (2 g/Kg ip). Blood samples were taken by tail bleeding at 0, 30, 60, and 90 minutes after injection to determine plasma glucose concentration. The total area under the curve was calculated as mmol/L/90 min.

*Assessment of the Lipid Profile*. At the end of the experimental period blood samples were drawn from the animals, after fasting for 12 hours. Total plasma cholesterol, HDL-cholesterol, and triglycerides were assessed using photocolorimetric enzymatic methods (Wiener Lab., Rosario, Argentina). Data are expressed in mmol/L.

### 2.4. Oxidative Stress Determinations

#### 2.4.1. Measurement of Plasma Thiobarbituric Acid-Reactive Substances (TBARS)

In order to demonstrate the effect of increased oxidative stress at the vascular level, plasma lipid peroxidation was assessed by TBARS concentration. This method was based on the reaction between plasma malondialdehyde, a product of lipid peroxidation, and thiobarbituric acid, as has been previously described [13]. No correction for sample protein content was necessary because of the nature of sample [14].

### 2.5. Measurement of Vascular NAD(P)H-Oxidase Activity

The lucigenin-derived chemiluminescence assay was used to determine NAD(P)H-oxidase activity in a segment of thoracic aorta, as previously described [14]. To assess NAD(P)H-oxidase activity, NADPH (500 *μ*mol/L) was added, and chemiluminescence was immediately measured in a liquid scintillation counter (LKB Wallac Model 1219 Rack-Beta Scintillation Counter, Finland) set in the out-of-coincidence mode. Time-adjusted and normalized-to-tissue-weight scintillation counters were used for calculations. Measurements were repeated in the absence and presence of diphenyleneiodnium (DPI) (10-6 mol/L), which inhibits flavin-containing enzymes, including NAD(P)H oxidase [15, 16].

### 2.6. eNOS Activity in Homogenates of Cardiac and Arterial Tissue

The activity of Ca^2+^/calmodulin-dependent endothelial nitric oxide synthase, (eNOS) was measured in mesenteric arteries homogenates and in left ventricle cardiac tissue, by conversion of L-[3H]arginine into L-[3H]citrulline. Values were corrected according to protein contents in the homogenates (Bradford method) and to incubation time and are expressed as dpm/mg protein/min. The material obtained from each animal was processed independently [17].

### 2.7. Relative Heart Weight

In order to evaluate cardiac hypertrophy, we measured relative heart weight (RHW). Briefly, heart was separated from the great vessels, dropped into a buffered saline solution (PBS), blotted with tissue paper to remove blood, and weighed. Total heart weight was corrected according to the ratio between heart weight (milligrams) and 100 grams of the total body weight before killing.

### 2.8. Measurement of High-Sensitive C-Reactive Protein (hs-CRP) Concentration

Plasma hs-CRP concentrations were measured using a turbidimetric assay (Bayer Advia 1650, AG Leverkusen). Data are expressed in mg/L.

### 2.9. Tissue Preservation

Tissue samples for histopathology were processed as has been previously reported [15]. Samples from all rats were used for these observations. Anesthetized animals were briefly perfused with PBS (298 mOsmol/Kg H_2_O, pH 7.40, 4°C) to clear out the blood. Mesenteric arteries were perfused in vivo with the same solution through the mesenteric artery during 5 min. For histological studies, arteries were also perfused with 4% paraformaldehyde solution for 10 min and fixed by paraffin. Five *μ*m-thick tissue slices were transversely cut across the mesenteric tissue on a microstate (Microm HM, Germany) and processed for histological studies. Similar procedure was applied for heart tissue preservation, by aortic retrograde perfusion. 

### 2.10. Quantitative Histomorphometry to Determine Cardiac Hypertrophy

Histomorphological analyses were conducted on slices from the outer (free) wall of the left ventricle (LV) of the heart. Estimations of cardiomyocyte area were made from sections stained with Masson trichrome solution. Areas with transverse sections of myofibers were selected. The contour of the fibers was then drawn manually. Total myocardiocyte area was expressed as square micrometer (*μ*m^2^). 

### 2.11. Arterial Structure

Changes in the structure of arterial walls were assessed by measuring the media layer in mesenteric arteries. Dissected mesenteric vascular beds were fixed in 10% formaldehyde, dehydrated, embedded in paraffin, and later cut in microtome. The slices were dyed and examined as has been previously described [15]. Nontransverse sectioned arteries were excluded from investigation. The lumen to media ratio (i.e., internal diameter to medial thickness) (L/M) was then calculated. Fifty slices from each animal were processed and analyzed to obtain an average value for each rat. Average values were then used for final analysis.

### 2.12. SDS-PAGE and Immunoblot Analysis

Mesenteric tissue was washed in PBS and proteins extracted in cold 20 mM Tris-HCl, pH 7.4, 150 mM NaCl, 10% glycerol, 1% Triton X-100, and a protease inhibitor mixture (P2714, Sigma). After sonication for 15 s (3 times with 10 s intervals) and extraction for 30 min at 4°C, sample extracts were clarified by centrifugation at 14,000 ×g for 20 min and used immediately or stored at −20°C. Proteins were separated on 10% polyacrylamide slab gels and transferred to 0.22 *μ*m nitrocellulose membranes (GE, Germany). Nonspecific reactivity was blocked by incubation for 1 h at room temperature in 5% nonfat dry milk dissolved in washing buffer (PBS, pH 7.6, 0.2% Tween 20). Blots were incubated with anti-p65 and anti-VCAM-1 antibodies (0.2 *μ*g/mL in blocking solution) for 60 min at room temperature. Horseradish peroxidase-conjugated goat anti-rabbit-IgG and swine anti-goat-IgG dissolved in blocking buffer were used as secondary antibodies (0.25 *μ*g/mL, 45 min at room temperature). Excess first and second antibodies were removed by washing 5 times for 5 min in blocking solution. Detection was accomplished with enhanced chemiluminescence system (ABC, Dako System) and subsequent exposure to Kodak X-AR film (Eastman Kodak) for 5–30 s. 

### 2.13. Immunohistochemistry and Digital Confocal Microscopy (IHC)

#### 2.13.1. Determination of Transcription Factors (WB)

Rabbit anti-rat NF-*κ*B p65 subunit [Rel  A], C-terminus antibody, was obtained from Millipore International Inc. (Amsterdam, The Netherlands) (AB1604b), and goat anti-rat VCAM-1 (C-19) antibody was obtained from Santa Cruz Biotechnology Inc. (Santa Cruz, CA, USA) (sc-1504). Tissue sections were cut at 3 *μ*m thickness from paraffin-embedded blocks. Deparaffinized sections were used to determine inflammatory response. Tissue was permeabilized in 1% Triton X-100 for 15 min, rinsed well with PBS, and blocked with sterile filtered 10% normal rabbit serum for 20 min. All antibody solutions were microfuged for 20 min before use. The antibodies were 1 : 1000 diluted. Primary incubations lasted 1 hour at 21-22°C, followed by extensive washes in PBS with Triton X-100, six times for 5 min each. Secondary antibodies, anti-rabbit IgG TR and anti-goat IgG FITC (Sigma-Aldrich), were diluted in PBS alone in compliance with the manufacturer's instructions. 

Images were collected with Nikon EZ-C1 3.00 software on a Nikon Diaphot TMD microscope equipped for fluorescence with a xenon lamp and filter wheels (Sutter Instruments, Novato, CA, USA), fluorescent filters (Chroma, Brattleboro, VT, USA), cooled charge-coupled device camera (Cooke, Tonawanda, NY, USA), and stepper motor (Intelligent Imaging Innovations, Inc., Denver, CO, USA). Multifluor images were merged, deconvolved, and renormalized using EZ-C1 3.00 Thumbnail software.

#### 2.13.2. Determination of Matrix Metalloproteinases

Anti-MMP-2 was obtained from Chemicon International Inc. (MAB3308), and anti-MMP-9 antibody was obtained from Chemicon (MAB3309). Tissue sections were cut at 5 *μ*m thickness from paraffin-embedded blocks. Deparaffinized sections were used to determine inflammatory response. Tissue was permeabilized in 1% Triton X-100 for 15 min, rinsed well with PBS, and blocked with sterile filtered 10% normal rabbit serum for 20 min. All antibody solutions were microfuged for 20 min before use. The antibodies were diluted 1 : 500. Primary incubations were done for 1 hour at 21-22°C, followed by extensive washes in PBS with Triton X-100, generally six times for 5 min each. Secondary antibodies, Cy5 and FITC IgG (Sigma-Aldrich), were diluted in PBS alone in compliance with the manufacturer's instructions. 

Images were collected with Nikon EZ-C1 3.00 software on a Nikon Diaphot TMD microscope equipped for fluorescence with a xenon lamp and filter wheels (Sutter Instruments, Novato, CA, USA), fluorescent filters (Chroma, Brattleboro, VT, USA), cooled charge-coupled device camera (Cooke, Tonawanda, NY, USA) and stepper motor (Intelligent Imaging Innovations, Inc., Denver, CO, USA). Multi-fluor images were merged, deconvolved, and renormalized using EZ-C1 3.00 Thumbnail software.

*Activity of Matrix Metalloproteinases 9 and 2 (Collagenases).* A sample of mesenteric tissue homogenates is obtained using 50 ug of total protein; subsequently a polyacrylamide gel co-polymerized with gelatin was used. Composition is as follows: 2880 uL bidistilled water (BD, milliQ), 800 uL gelatin 10 mg/mL (Sigma, USP, etc.) (end conc: 1 mg gelatin/mL of gel), 2160 uL acrylamide/bis 30%, 2160 uL Tris-Cl 1.5 M pH 8.8, 80 uL SDS 10% (Sigma), 80 uL of ammonium persulfate 10%, and 6 uL TEMED. A third of the sample buffer was used based on the total sample volume with proteins. The run was stopped when the phenol red reached the lower edge of the gel and was starting to overflow it. Then, the gels were washed to remove the SDS. The gels were washed in 30–50 mL of Triton X-100 2.5% for 20–30 min with continuous agitation. Incubation in 50 mL (per gel) of this solution at 37°C for 12 h approximately Afterwards the gels were stained with Coomassie blue R-250 for 12 h. For higher contrast it was used at a concentration of 0.5% (w/v) instead of 0.1%. Gels were decolorated with solution of methanol, acetic acid, and water. Metalloprotease activity was evaluated based on mean optical density of light bands on a dark blue background.

### 2.14. Reagents

The drug Lumiracoxib in pure state was provided by Novartis Basel.

Unless otherwise noted, reagents were purchased from Sigma Chemical Co, MO, USA.

### 2.15. Statistical and Data Analysis

Data are expressed as mean ± SEM. The statistical significance of data comparison between all groups was assessed by one-way ANOVA followed by Bonferroni posttest. A two-sided *P* value of less than 0.05 was considered significant.

## 3. Results

Levels of systolic blood pressure increased gradually throughout the entire experimental period in animals of groups FFR, SHR, and FFHR, reaching significant differences with respect to the control group at the end of the protocol (Table 1). Chronic treatment with L significantly decreased TA values in groups FFR, FFHR, and SHR but not to the values of the control group (Table 1).

Furthermore, FFR and FFHR groups are characterized as a model of metabolic syndrome according to the increase of the HOMA index, fasting blood glucose, triglycerides, decreased HDL-cholesterol, and arterial hypertension. Animals in the experimental groups FFR and FFHR which received L did not have significantly modified values of fasting blood glucose, triglycerides, and HDL-cholesterol (Table 1).

Oxidative stress variables were evaluated in the four experimental models. NADPH oxidase activity increased significantly in all experimental models but did so exponentially in the FFHR model. On the other hand eNOS activity decreased significantly in the experimental models with fructose feeding (Table 1). Lipid peroxidation was evaluated from TBARS, which showed significant increases in all three experimental models, the FFHR model being the most affected. After chronic treatment with L, aortic NAD(P)H oxidase activity significantly decreased in groups FFR, SHR, and FFHR. Endothelial eNOS activity normalized in the experimental models FFHR and FFR after treatment with L, and these results were statistically significant.

In addition, cardiac remodeling was evaluated based on relative cardiac weight (RHW) (Table 1). Experimental models FFR, SHR, and FFHR showed cardiac remodeling, which decreased, after chronic treatment with L, in the experimental groups studied but only the FFHR group showed statistically significance. 

Vascular remodeling, as discussed previously, was evaluated based on the M/L ratio. In experimental models FFR, and FFHR a significant reduction of the media/lumen ratio was observed, demonstrating the presence of eutrophic remodeling of the mesenteric arteries studied. After chronic treatment with L, we demonstrated an increase of this variable in the three experimental models, which caused the remodeling to decrease. It should be noted that this increase in the remodeling index, although statistically significant, did not reach normal values, which is an important fact when analyzing the involvement of COX-2 in the pathophysiological mechanisms.

After demonstrating the presence of cardiovascular remodeling in these experimental models, we studied the presence of inflammatory markers.

At the systemic level, C-reactive protein (hsCRP) was evaluated; it showed significant differences among FFR and FFHR models, demonstrating the presence of inflammation in these groups. After treatment with a COX-2 specific antagonist, these variables significantly decreased in the three experimental models, reaching normal values and demonstrating the participation of this pathophysiological pathway in the systemic inflammatory process.

**Figure 1:**
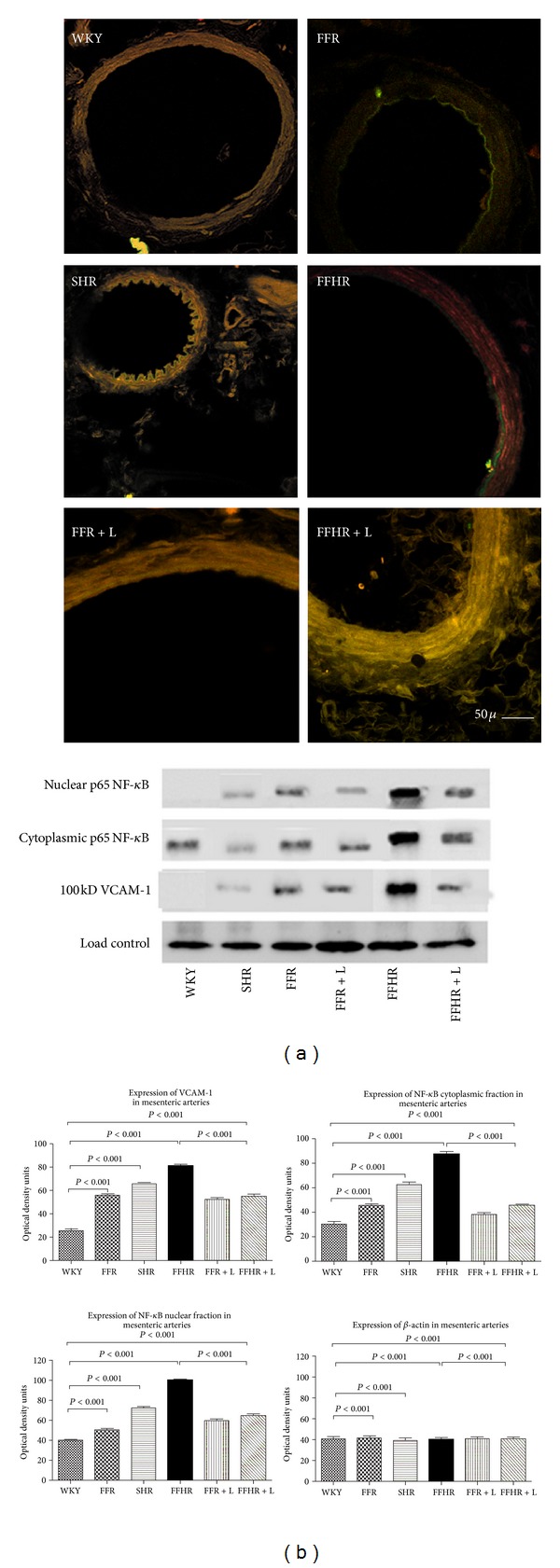
Cytoplasmatic and nuclear p-65 fraction of NF-*κ*B and VCAM-1 expression in mesenteric arteries by WB and IHC. up panel shows the WB representative membrane and which analyzed anti-VCAM-1-FITC and anti-p65-TRITC, the results were obtained by optic density of the bands revealed for each group. top panel shows microphotographs obtained by laser ICM 600x of mesenteric tissue.

At the vascular level, the expression of these proteins was assessed by Western blot as shown in Figure 1. In experimental models FFR, FFHR, and SHR the expression of these markers increased. After chronic treatment with L, the translocation of NF-*κ*B to the nucleus and the expression of VCAM-1 at the cellular membrane level were both reduced in the experimental models FFR and FFHR (Figure 1).

The expression of the aforementioned markers was studied by IFI; they were exposed in the vascular wall by laser colocalization microscopy. The experimental models studied showed different expression patterns of NF-*κ*B and VCAM-1. This result was previously reported by our laboratory [18]. Model FFHR presents an important expression of NF-*κ*B in the entire vascular wall (intima, media, and adventitia) as well as endothelial expression of VCAM-1, while in other models both markers were present only in the endothelium. Treatment with L significantly reduced the expression of these markers, as demonstrated by WB, although inflammation at the adventitia level was higher in the FFHR model (Figure 1.)

**Figure 2:**
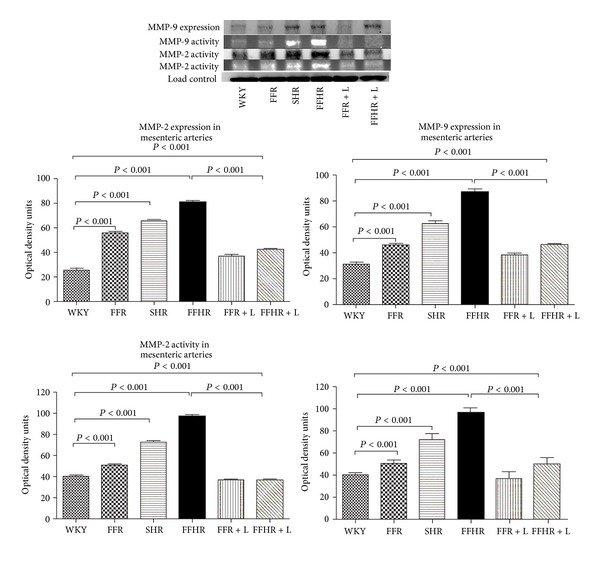
A representative polyacrylamide gel for Western blot and zymography for collagenases (MMP-2 and MMP-9). This image showed activity and expression of collagenases contrasted for each experimental group. The picture below shows the bar graph with statistical analysis.

**Figure 3:**
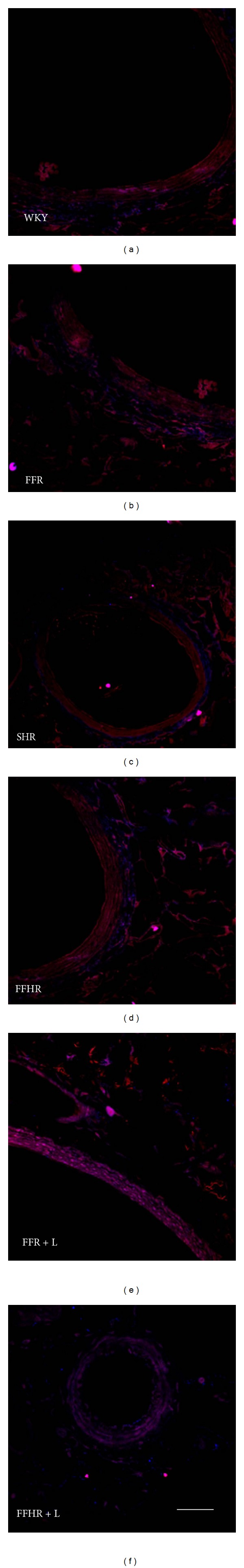
Representative figure showing the expression of MMP-2 (blue: Cy5) and MMP-9 (red: TRICT) merge mode in mesenteric arteries. You can see an increased level of tagging in adventitia for MMP-2 and MMP-9 on experimental models with vascular remodeling: SHR, FFR, and FFHR. These changes are reversed after administration of L. Microphotographs obtained by laser ICM 600x of mesenteric tissue.

We also analyzed two markers of vascular remodeling, MMP-2 and MMP-9. The expression was analyzed using WB and the activity by zymography. In models FFR and SHR the activity and the expression of these MMPs increased moderately, while the FFHR model presents maximum activity and expression of both collagenases (Figure 2). Chronic treatment with L significantly reduced the activity and expression of the MMPs, although in the FFHR model this decrease did not reach the values of the control group and persisted primarily at the adventitial level. Figure 2 shows a representative image of the gels and the statistical analysis.  Figure 3 shows expression by IFI of representative photomicrographs of these antibodies, with the aim of obtaining a vascular level location of this expression. MMP-2 and MMP-9 are observed predominately at the adventitia level.

This finding provides further evidence of the importance of this vascular layer present in vascular remodeling of metabolic syndrome.

## 4. Conclusions

The most important finding of this study was the demonstration that ciclioxigenasa-2 participates in the cardiovascular remodeling associated with metabolic syndrome and the reversal of this syndrome after chronic treatment with a specific antagonist, lumiracoxib (L).

We demonstrate that chronic treatment with L did not modify the metabolic variables associated with metabolic syndrome of models FFHR and FFR. It partially modified the oxidative stress variables but reduced total lipid peroxidation as demonstrated by TBARS, reducing vascular damage and probably reducing the expression of redox-sensitive genes such as NF-*κ*B, an important initiator of inflammation.

Both vascular and cardiac remodeling showed significant differences after treatment with L, mainly in the FFHR model. This was also evidenced in microscopic sections of mesenteric arteries showing less inflammation and decreased expression of extracellular matrix metalloproteinases.

The initiation of the atherosclerotic process is associated, according to some authors, with the presence of endothelial dysfunction (as demonstrated in this model) and local inflammation in a first stage, after activating macrophages that synthesize IL-6, which interacts with hepatocytes and initiates the release of acute phase reactants such as CRP. NF-*κ*B is a redox-sensitive transcription factor of great potency for initiating and perpetuating the inflammatory response; its most important transcriptional product at the vascular level is VCAM-1, a marker both of inflammation and of endothelial dysfunction according to several authors. The number of these two molecules increased in the entire vascular wall in the FFHR model and only in the intima layer in FFR and SHR models, as if they were intermediate products of WKY rats and FFHR. Epigenetics may explain the differential expression in this model, as fructose is a hidden genes demethylation in SHR that culminates, at the vascular level, showing a completely swollen vessel. Chronic treatment with L altered the expression of inflammatory molecules in the models that received fructose, although at a microscopic level there remains a residue of inflammation in the adventitia.

The remodeling of the vessels can demonstrate that collagenases, which perform the work “softening” the matrix, are present in increased numbers [19]. Although the fact that they have increased does not imply the presence of remodeling, for that it should be demonstrated that their activity has also increased and that is why zymography used. Both MMP-2 and MMP-9 increased in the models studied. Chronic treatment with L modified the expression and activity of MMP-2 and MMP-9, increasing at a microscopic level the media/lumen ratio and thereby improving the vascular microenvironment.

Other authors have also shown the involvement of COX-2 in vascular pathophysiology; Dinarello et al. suggest that a detrimental action of COX-2 has also been described in hypertensive patients and represents one mechanism whereby COX-2 may promote atherosclerosis; also they find that vascular COX-2 downregulation represents a prominent mechanism whereby statins may attenuate the development of the atherosclerotic process in normocholesterolemic hypertensive patients [20].

As a final conclusion, cyclooxygenase actively participates in inflammation process and in the remodeling of the vessels, being an important factor that in the future will allow us to use these results to pharmacologically improve these antagonists so that they can be used in clinical practice as therapeutic targets or as adjuvants in more complete drug therapies the way monoclonal antibodies are used now [21].

## Figures and Tables

**Table 1:** The above values correspond to metabolic and cardiovascular variables.

Variables	W	FFR	SHR	FFHR	FFHR + L
SBP (mmHg)	118 ± 0.8	140 ± 1.8*	172 ± 2.0^∗*∧*^	182 ± 1.1^∗*∧*#^	165 ± 0.9^∗*∧*†^
HOMA (*μ*U/mL insulin × mmol/L glucose)/22.5	4.22 ± 1.1	11.9 ± 1.3*	8.1 ± 2.2*	15.1 ± 2.5*	13.2 ± 2.1*
Fast glucemia (mmol/L)	4.0 ± 1.1	6.8 ± 1.3*	5.2 ± 1.3*	6.92 ± 2.1*	6.5 ± 2.1*
Tryglycerides (mg/dL)	72.5 ± 1.9	109 ± 1.8*	115 ± 2.4*	149 ± 2.2^∗*∧*#^	140.9 ± 2.4*
Relative heart weight (mg/100 g corporal weight)	229 ± 2.5	302 ± 2.1*	425 ± 4.4*	475 ± 2.6^∗*∧*#^	389 ± 2^∗*∧*†^
Vascular NAD(P)H-oxidase activity (cpm/mg)	14.5 ± 3.3	68 ± 1.4*	149 ± 2.6*	366 ± 12^∗*∧*#^	197 ± 2.3^∗*∧*#†^
TBARS (mmol/L)	39 ± 3.2	118 ± 5.4*	110 ± 3.9*	171 ± 2.6^∗*∧*#^	101 ± 2.7^∗*∧*#†^
Arterial eNOS activity (dpm·mg P/min)	82.0 ± 2	62 ± 1.5*	81.9 ± 2.6	50.6 ± 1.9^∗*∧*#^	71.4 ± 1.1^∗*∧*#†^
L/M relationship	13.1 ± 1.4	9.5 ± 1.2*	8.9 ± 2.1*	7.4 ± 1.2^∗#^	11.5 ± 1.1^∗*∧*#†^
PCRus	1.18 ± 0.1	3.2 ± 0.3*	4.5 ± 0.1*	6.7 ± 0.2^∗*∧*#^	1.1 ± 0.2^∗*∧*#^

*P < 0.001 versus WKY; ^*∧*^ *P* < 0.001 versus SHR; ^#^ *P* < 0.01 versus FFR. ^†^versus FFHR.
